# Microbial Antigen-Presenting Extracellular Vesicles Derived from Genetically Modified Tumor Cells Promote Antitumor Activity of Dendritic Cells

**DOI:** 10.3390/pharmaceutics13010057

**Published:** 2021-01-04

**Authors:** Tomoko Ito, Kikuya Sugiura, Aya Hasegawa, Wakana Ouchi, Takayuki Yoshimoto, Izuru Mizoguchi, Toshio Inaba, Katsuyuki Hamada, Masazumi Eriguchi, Yoshiyuki Koyama

**Affiliations:** 1 Japan Anti-Tuberculosis Association, Shin-Yamanote Hospital, Tokyo 189-0021, Japan; meriguchi@shinyamanote.jp; 2 Department of Advanced Pathobiology, Graduate School of Life and Environmental Sciences, Osaka Prefecture University, Osaka 598-8531, Japan; sugiura@vet.osakafu-u.ac.jp (K.S.); sc13421@yahoo.co.jp (A.H.); wkna.o.dkrjg@gmail.com (W.O.); inaba@vet.osakafu-u.ac.jp (T.I.); 3 Department of Immunoregulation, Institute of Medical Science, Tokyo Medical University, Tokyo 160-8402, Japan; yoshimot@tokyo-med.ac.jp (T.Y.); mizoizu0403@yahoo.co.jp (I.M.); 4 Department of Clinical Oncology, School of Medicine, Toho University, Tokyo 143-8541, Japan; katsuyuki.hamada@med.toho-u.ac.jp

**Keywords:** extracellular vesicles, cancer immunotherapy, neoantigens, ESAT-6, dendritic cells

## Abstract

Tumor-derived extracellular vesicles (EVs), as tumor vaccines, carry tumor-associated antigens (TAAs), and were expected to transfer TAAs to antigen-presenting cells. However, treatment with tumor-derived EVs exhibited no obvious antitumor effect on the established tumors, likely due to their immuno-suppressive functions, and also to the poor immunogenicity of TAAs. In order to improve the immune stimulating properties, EVs expressing a highly immunogenic bacterial antigen, 6 kDa early secretory antigenic target (ESAT-6), from Mycobacterium tuberculosis were prepared by genetically modifying the parent tumor cells with a plasmid coding for ESAT-6. Cultured B16 tumor cells were transfected with a ternary complex system consisting of pDNA, polyethylenimine (PEI), and chondroitin sulfate. The cells that were transfected with the ternary complex secreted EVs with a higher number of ESAT-6 epitopes than those transfected by a conventional DNA/PEI binary complex, due to the low cytotoxicity, and durable high expression efficiency of the ternary complex systems. The EVs presenting the ESAT-6 epitope (ESAT-EV) were collected and explored as immune modulatory agents. Dendritic cells (DCs) were differentiated from mouse bone marrow cells and incubated with ESAT-EV. After incubating with the EVs for one day, the DCs expressed a significantly higher level of DC maturation marker, CD86. The DCs treated with ESAT-EV showed a significantly improved antitumor activity in tumor-bearing mice.

## 1. Introduction

Extracellular vesicles (EVs) including exosomes that are derived from tumor cells are known to carry an array of tumor-associated antigens (TAAs). Initially, they were expected to be ideal candidates for use in tumor vaccines to transfer the TAAs to antigen-presenting cells (APCs) [1]. The polyepitopic antigen presentation on tumor-derived EVs could potentially elicit appropriate immune responses against tumors [2]. In some cases, tumor-derived EVs offered effective prophylactic protection against tumor challenge. Vaccination with the exosomes secreted from L1210, a mouse lymphocytic leukemia cell line, has been found to prevent tumor formation and prolong survival [3]. The administration of exosomes from brain tumor cell lines into syngeneic animals has also resulted in the rejection of subsequent challenges of the same tumor, inducing both humoral and cellular immune responses [4]. However, despite hoping that tumor-derived EVs would be promising immunotherapeutic tools to induce potent anti-tumor immune responses, treatment with the EVs after tumor implantation has not been found to exert a clear antitumor effect. One reason, for the reduced effectiveness of tumor-derived EVs for tumor regression, is their immune suppressive functions. Tumor-derived EVs are now known to carry a cargo of various immunosuppressive molecules, including inhibitory cytokines, such as IL-10 and TGF-β1, death receptor ligands, such as FasL or TRAIL, and checkpoint receptor ligands, such as PD-L1 [5]. As a result, the tumor-derived EVs perform dual roles in both immune stimulation and suppression [6].

Another reason for the low antitumor efficacy of the tumor-derived EVs is the poor immunogenicity of TAAs. TAAs are generally poorly antigenic and may not be readily recognized as danger signals by dendritic cells (DCs) [7,8]. TAAs can be classified into the following two broad categories: (i) shared antigens that are overexpressed in tumor cells and also present in normal tissues and (ii) cancer-specific neoantigens that arise from somatic mutations in tumor cells. On the one hand, tumor cells are variants of a patient’s own normal cells. Therefore, it is difficult for shared antigens to become the target of immunotherapy, and some degree of immune tolerance may already be established against them. This results in the low efficacy of tumor-derived EVs in cancer immunotherapy for patients whose tumor cells only have shared antigens. On the other hand, neoantigens are antigens that the immune system meets for the first time. When the neoantigen has epitopes with high affinity to major histocompatibility complex (MHC) molecules, the epitope/MHC molecule complex is formed. The MHC molecule with bound epitope moves to the cell surface and is recognized by appropriate T-cells [9,10]. Immunotherapy should, thus, be effective only for patients whose tumor cells have highly immunogenic neoantigens that are immune targetable. Snyder et al. characterized the exomes of malignant melanoma cells from patients that had received immunotherapy blocking CTLA-4 (cytotoxic T-lymphocyte-associated protein 4), which functions as an immune checkpoint and downregulates immune responses. They reported that some highly antigenic neoepitopes were present in the patients with a prolonged clinical benefit, but absent in patients without this benefit [11]. Tumor-specific neoantigens that are recognized by the immune system as a foreign “danger signal” are essential to elicit a host immune response against tumors. However, only a small minority of patients have such effective neoantigens that can be recognized as an immune target and elicit antitumor immune response. The difficulty associated with the treatment of tumors without effective neoantigens is a recently recognized issue and represents a major limitation of immunotherapy [12].

Tumor-derived EVs, in most cases, carry only poorly immunogenic nonmutated shared antigens. This can be attributed to the low immunity-inducing ability of EVs. In order to improve the immune stimulating properties of EVs from common tumor cells without neoantigens, we developed “artificial neoantigen-presenting EVs” by transforming parent tumor cells with a plasmid to express highly immunogenic bacterial antigens. In a previous study, we transfected cultured B16 melanoma cells to express 6-kDa early secreted antigenic target (ESAT-6) protein from Mycobacterium tuberculosis. The ESAT-6 protein was produced in the transfected tumor cells, degraded in the proteasome into epitopes, and finally presented on the cell surface as “artificial neoepitopes” in the context of MHC class I. Then, those cells secreted the EVs to which the MHC Class I/ESAT-6 epitope complex was transferred. We isolated the secreted EVs presenting ESAT-6 epitopes (ESAT-EV) from the culture media. The ESAT-EV exhibited significant tumor growth inhibition in mice bearing parent B16 tumors (Figure S1, Supplementary Materials). The lymphocytes from mice treated with ESAT-EV secreted a significantly higher level of IFN-γ when co-cultured with B16 cells [13].

The antitumor effect of the “artificial neoepitope-”presenting EVs is most likely mediated by antigen-presenting cells (APCs), which capture the EVs and recognize the ESAT-6 epitopes as an exogenous ‘‘danger signal’’. Macrophages may play a role in enhancing the immune responses by secreting cytokines. DCs are also thought to play a key role in inducing antitumor immunity. DCs can take up the EVs, recognize the ESAT-6 epitopes, and undergo maturation. Then, the “artificial neoepitopes” are presented to T cells, together with the “tumor antigen epitopes”, and induce a cellular immunity to ESAT-6, as well as against weak immunogenic TAAs. In this study, the DC activation ability of the artificial neoepitope-presenting EVs was evaluated by analyzing co-stimulatory molecule presentation in EV-stimulated DCs. Furthermore, the antitumor efficacy of the DCs stimulated by the EVs was studied in a syngeneic mouse model.

## 2. Materials and Methods

### 2.1. Materials and Mice

Polyethylenimine “Max” (MW 40,000 in a hydrochloride salt form, comparable to MW 25,000 in a free base form) was purchased from Polyscience, Inc. (Warrington, PA, USA). Chondroitin sulfate sodium salt from shark cartilage (MW 10,000) was supplied by Seikagaku Corp. (Tokyo, Japan). Total exosome isolation (from cell culture media) and recombinant murine granulocyte macrophage colony-stimulating factor (GM-CSF) were purchased from Thermo Fisher Scientific Inc. (Waltham, MA, USA) and PeproTech, Inc. (Rocky Hill, NJ, USA), respectively. The plasmid harboring the ESAT-6 gene was prepared by Takara Bio Inc. (Shiga, Japan) by inserting the gene into a pcDNA3.1 vector with a Kozak sequence (GCCACC). The plasmid was amplified by AMBiS Corporation (Okinawa, Japan). The ESAT-6 protein and lipopolysaccharides were obtained from Novus Biologicals, LLC (Centennial, CO, USA) and Sigma-Aldrich (St. Louis, MO, USA), respectively. Rabbit anti-ESAT-6 polyclonal antibody was purchased from Bioss Antibodies Inc. (Woburn, MA, USA). Monoclonal antibodies (mAbs), FITC-labeled goat anti-rabbit IgG antibody, anti-mouse CD16/32 (2.4G2), PE-Cy7-labeled anti-mouse CD86 (PE-Cy7-CD86), PE-Cy7-labeled IgG1, k (Cont IgG), and APC-Cy7-labeled anti-mouse CD11c (APC-Cy7-CD11c) were obtained from Biomedical Technologies, Inc. (Stoughton, MA, USA) and BioLegend (San Diego, CA, USA). Male C57BL/6CrSlc mice (5 or 6 weeks old) and female C57BL/6J mice (7 weeks old) were purchased from Japan SLC, Inc. (Shizuoka, Japan) and Sankyo Labo Service Corporation, Inc (Tokyo, Japan), respectively. All animal studies were carried out in accordance with the guidelines of Osaka Prefecture University or Tokyo Medical University and were approved by the ethics committee of each university (Osaka Prefecture University: Approval No. 28-23 on 1 April 2017 and Approval No. 29-16 on 1 April 2018; Tokyo Medical University: Approval No. H31-0041 on 5 March 2019).

### 2.2. Preparation of Extracellular Vesicles (EVs)

EVs derived from tumor cells that had been transfected with the ESAT-6 gene were prepared, as described in a previous study [13]. Briefly, B16 mouse melanoma cells were seeded onto 24-well plates at 5.0 × 10^4^ cells per well and cultured for 2 days in Eagle’s minimum essential medium (EMEM) supplemented with 10% fetal bovine serum (FBS), penicillin G sodium (100 unit/mL), and streptomycin sulfate (0.1 mg/mL). Then, the primary growth medium was replaced with 500 μL of fresh EMEM with 10% exosome-depleted FBS (Thermo Fisher Scientific Inc.), and the antibiotics. The DNA ternary complex was obtained by mixing an aqueous solution of pDNA encoding the ESAT-6 gene with those of PEI and CS, in this order, at the charge ratio of 1:12:8, and added to the cells (1.5 μg DNA/well). After incubation for 5 h at 37 °C in a 5% CO_2_ incubator, the medium was replaced with 1 mL of fresh medium containing 10% exosome depleted FBS and the antibiotics. The cells were incubated for an additional 3 days. Then, the cell culture medium was collected and centrifuged at 3000× *g* for 15 min to remove cells and debris. The supernatant was transferred and mixed with a half volume of total exosome isolation reagent. The mixture was incubated at 4 °C overnight, and then centrifuged at 10,000× *g* for 1 h, at 4 °C, to precipitate the EVs presenting ESAT-6 epitopes (ESAT-EV). The precipitated EVs were re-suspended in Opti-MEM I Reduced Serum Medium (Thermo Fisher Scientific Inc., Waltham, MA, USA) and stored at 4 °C, up to 7 days before use. The transfection with conventional DNA binary complex was carried out in a similar manner with DNA/PEI mixture (1:12 in charge). EVs not presenting ESAT-6 epitopes (Naive EV) were also prepared similarly, as a reference from B16 cells without ESAT-6 transfection. The particle number of the isolated EVs was evaluated using an EXOCET Exosome Quantitation Assay Kit (System Biosciences, Palo Alto, CA, USA).

### 2.3. Quantitation of 6 kDa Early Secretory Antigenic Target (ESAT-6) Epitopes on ESAT-6 Epitope-Presenting EVs (ESAT-EV)

T-cell immunoglobulin domain and mucin domain-containing protein 4 (Tim-4), which specifically binds to phosphatidylserine, was immobilized in a 96-well plate using a PS Capture™ Exosome ELISA Kit (FUJIFILM Wako Pure Chemical Corporation, Osaka, Japan). The EVs prepared above (1.9 × 10^8^ in 10 μL) were added to the plate and allowed to stand for 1 h at room temperature. After washing 3 times with 300 μL of washing buffer from the PS Capture™ Exosome ELISA Kit, 50 ng of rabbit anti-ESAT-6 antibody in 100 μL of the washing buffer was added to each well. After standing for 1 h at room temperature, the wells were washed three times with 300 μL of the washing buffer, followed by the addition of FITC-labeled goat anti-rabbit IgG antibody (50 ng, in 100 μL washing buffer). The mixture was incubated for 1 h, and the wells were again washed three times with 300 μL of the washing buffer. The washing buffer (100 μL) was added to the wells, and the fluorescence intensity was measured using a microplate reader (XFluor4GENiosPro; TECAN, Zürich, Switzerland) with an excitation wavelength of 485 nm and an emission wavelength of 520 nm.

### 2.4. Size Distribution of the EVs

The size distribution of the EVs derived from the transfected or non-transfected B16 cells was evaluated with a NanoSight LM10 (Malvern Panalytical, Ltd., Malvern, UK) by a commissioned test of Cosmo Bio. Co. Ltd.

### 2.5. Preparation of Bone Marrow-Derived Dendritic Cells (DCs)

The femurs were removed from C57BL/6 mice, and the bone marrow was flushed with RPMI 1640, supplemented with 10 mM HEPES/NaOH and the antibiotics. The bone marrow cells were centrifuged at 1500 rpm for 3 min, and the precipitation was mixed with 0.5 mL of red blood cells lysis buffer (155 mM NH_4_Cl, 12 mM NaHCO_3_, and 0.1 mM EDTA). After 4 min, RPMI 1640 supplemented with 0.1 mM 2-mercaptoethanol (2-ME), 10% FBS, and the antibiotics were added, and the mixture was centrifuged at 1500 rpm for 3 min. The precipitation was re-suspended in RPMI 1640 with 2-ME, FBS, and the antibiotics, and then filtered through a nylon mesh. The resulting filtrate was centrifuged at 1500 rpm for 3 min, and the precipitated monocytes (3.0 × 10^6^) were cultured in a 10 cm dish with 8 mL of RPMI 1640 containing 160 ng of GM-CSF supplemented with 2-ME, FBS, and the antibiotics. The bone marrow cells were incubated for 6 days, with half of the medium replaced with fresh GM-CSF containing RPMI 1640 medium on Day 3. The cells were collected, and CD11c-possitive cells were selected using a magnetic-activated cell sorting (MACS) system (autoMACS Pro Separator; Miltenyi Biotec GmbH, Bergisch Gladbach, Germany) using anti-CD11c magnetic beads (Miltenyi Biotec GmbH, Bergisch Gladbach, Germany). Then, the isolated cells were subjected to a flow cytometric analysis. For the in vivo antitumor activity evaluation, the bone marrow cells were collected from the femurs, humeri, and tibias of C57BL/6 mice, and 1.0 × 10^7^ of the cells were cultured in 10 mL of RPMI 1640 containing 100 ng of GM-CSF, 10% FBS, and the antibiotics. Incubation was continued in the medium containing GM-CSF for 6 days, replacing the whole medium with fresh RPMI 1640 medium containing GM-CSF every 2 days. The differentiated DCs were collected and used in the antitumor efficacy experiments without further purification.

### 2.6. Flow Cytometric Analysis of DCs

DCs were prepared as described above and disseminated on 96-well plates at a density of 5 × 10^4^ cells per well, and then co-incubated with 1 × 10^10^ particles of ESAT-EV at the final concentration of 5 × 10^10^ particles/mL. The effect of Naive EV, or a mixture of Naive EV and 30 ng of ESAT-6 protein was also examined for comparison. After 1 day of co-incubation in 200 μL of Opti-MEM with 10% of exosome-depleted FBS and antibiotics at 37 °C with 5% CO_2_, the cells were harvested and centrifuged at 5000 rpm for 3 min. The precipitated cells were incubated with the 2.4G2 antibody to block non-specific Fc binding in FACS buffer (PBS with 2% FBS and 0.01% sodium azide) for 10 min on ice. After centrifugation at 5000 rpm for 3 min, the precipitated cells were treated for 15 min on ice with combinations of PE-Cy7-CD86 and APC-Cy7-CD11c, or Cont IgG and APC-Cy7-CD11c in FACS buffer. Then, flow cytometry was performed using a fluorescence activated cell sorter (FACS Canto II, BD Biosciences, Franklin Lakes, NJ, USA). The resulting data were analyzed using FlowJo Software (FlowJo LLC, Ashland, OR, USA).

### 2.7. 3H-Thymidine Incorporation by Lymphocytes from the ESAT-EV-Treated Mice

Leukocytes from the EV-treated mice were prepared, as described in a previous study [13]. Briefly, the EV suspension containing 3.8 × 10^9^ particles (in 150 µL PBS) was injected twice with an interval of 10 days into both foot pads of 5-week-old C57BL/6 mice (75 µL to each foot pad). As a control, 1 mg/mL of BSA solution was injected. The popliteal lymph nodes were collected 10 days after the second administration. The cells in the lymph nodes were dispersed in RPMI 1640 medium and seeded onto 96-well plates (2.0 × 10^5^ cells per well). The B16 melanoma cells were pretreated with Mitomycin C and added to the plates (1.0 × 10^4^ cells per well). They were co-cultured for 72 h in 0.2 mL of RPMI 1640 medium containing 10% FBS and the antibiotics, and then 3H-thymidine was added. After overnight incubation, the cells were harvested, and 3H-thymidine incorporation into the lymphocyte was measured using a liquid scintillation counter (LSC-6100, Hitachi, Tokyo, Japan).

### 2.8. Antitumor Effect of DCs Treated with ESAT-EV

The B16 melanoma cells were inoculated subcutaneously into the back of female C57BL/6 mice (1 × 10^6^ cells per mouse). The bone marrow-derived DCs (2 × 10^6^ cells), prepared above, were treated with 3 × 10^9^ particles of ESAT-EV or Naive EV in a 24-well plate with 250 μL of Opti-MEM supplemented with 10% of exosome-depleted FBS for 24 h at 37 °C with 5% CO_2_. After incubation, the DCs were washed and suspended with 250 μL of Opti-MEM, and then intratumorally injected three times at four-day intervals. The resulting change in the tumor size was monitored. The first injection was carried out after tumors grew to about 18 to 64 mm^3^ in volume. 

## 3. Results

### 3.1. EV Preparation by Cells Transfected with ESAT-6 Gene

**Figure 1:**
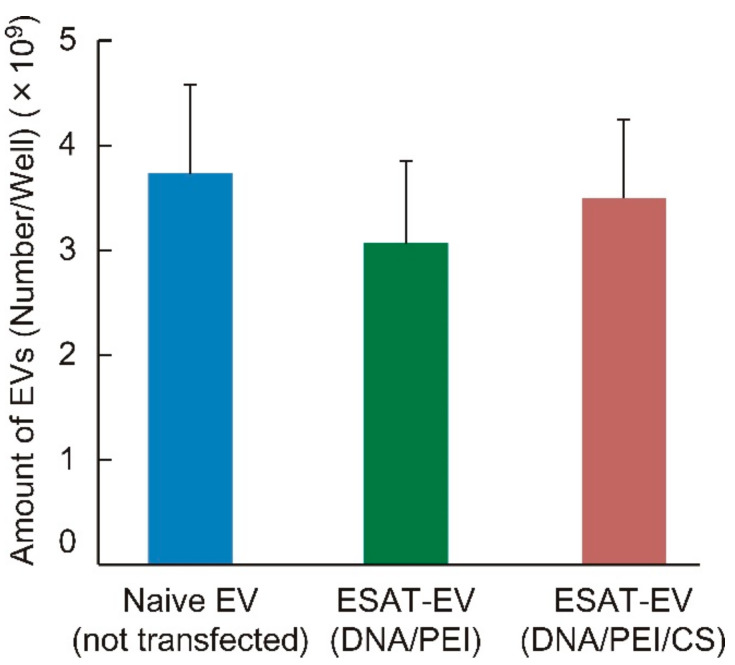
Extracellular vesicle (EV) production efficiency of genetically modified tumor cells. B16 melanoma cells were cultured in 24-well plates with or without the addition of the complex of pDNA coding for 6 kDa early secretory antigenic target (ESAT-6). DNA/polyethylenimine (PEI) binary complex, or DNA/PEI/chondroitin sulfate (CS) ternary complex, each containing 1.5 μg pDNA(ESAT-6)/well, were used to introduce the gene into the cells. At 3 days post transfection, the cell culture medium was collected, and EVs from the transfected cells (ESAT-EV) and those from the non-treated cells (Naive EV) were isolated by centrifugation, followed by precipitation with total exosome isolation reagent. The particle number of the isolated EVs was evaluated using an EXOCET Exosome Quantitation Assay Kit and are presented as the number of EVs in one well (*n* = 17, mean ± SD).

**Figure 2:**
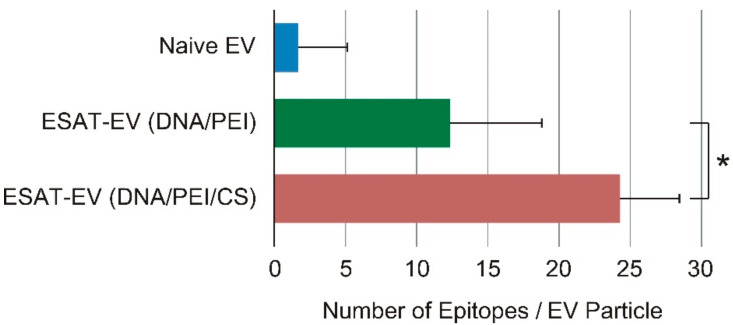
The number of ESAT-6 epitopes on EV particles secreted by the genetically modified tumor cells. EVs isolated from the culture medium of the transfected cells (ESAT-EV) by DNA/PEI binary complex, or DNA/PEI/CS ternary complex, and those from non-transfected cells (Naive EV) were trapped onto Tim-4-immobilized plate. The EVs were treated with rabbit anti-ESAT-6 antibody, and then FITC-labeled goat anti-rabbit IgG antibody. The amount of the antibody was estimated from the fluorescence intensity of the EVs using intact FITC-labeled goat anti-rabbit IgG antibody as standard (*n* = 4, mean ± SD, * *p* < 0.05).

**Figure 3:**
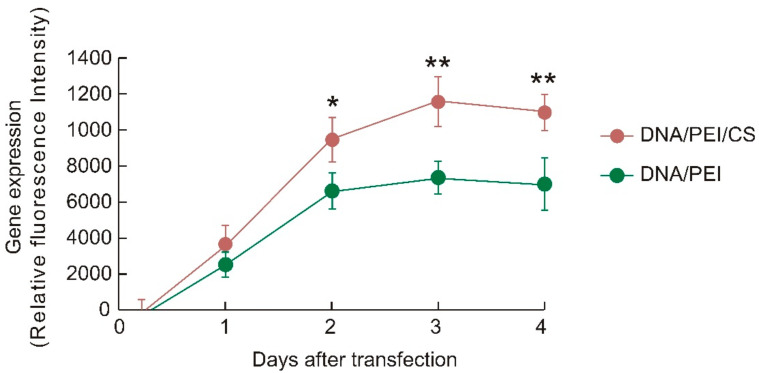
Gene expression efficiency in the cells transfected by the complex of pDNA coding for green fluorescent protein (GFP). The B16 melanoma cells were treated with pDNA(GFP)/PEI binary complex, or pDNA(GFP)/PEI/CS ternary complex. The fluorescence intensity of the wells was monitored for 4 days after transfection to evaluate the gene expression efficiency (*n* = 3, mean ± SD, * *p* < 0.05 and ** *p* < 0.01).

**Figure 4:**
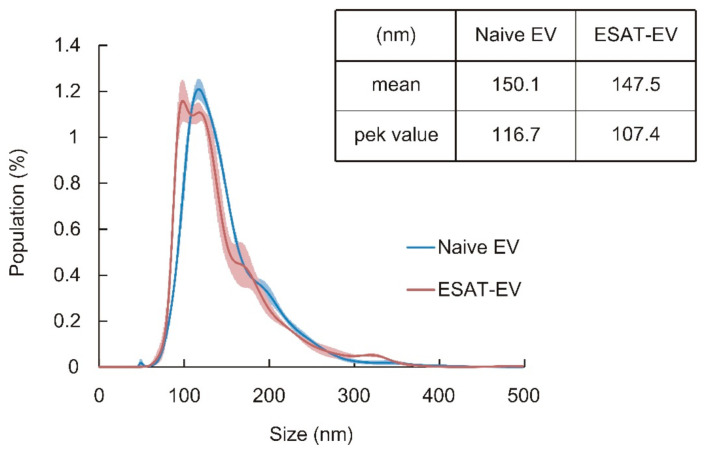
Particle size distribution, mean size, and peak value of EVs. EVs isolated from the culture medium of the B16 cells transfected by DNA/PEI/CS ternary complex (ESAT-EV) and those from the non-treated cells (Naive EV) were analyzed for their size and size distribution with NanoSight LM10. Blue and red lines denote the size distribution profiles of Naive EV and ESAT-EV, respectively.

EVs expressing an immune-stimulating antigen, ESAT-6 (ESAT-EV), were obtained from the culture medium of B16 cells that had been transfected with a plasmid DNA harboring the ESAT-6 gene. Transfection was carried out by a ternary complex system consisting of DNA, Polyethylenimine (PEI), and chondroitin sulfate (CS), or by a conventional binary complex without CS. EVs secreted from the cells without ESAT-6 transfection (Naive EV) were also prepared for comparison. On the one hand, transfection with the DNA/PEI/CS ternary complex only slightly affected the yield of EVs, and almost the same amount of ESAT-EV was obtained as Naive EV. On the other hand, transfection with the DNA/PEI binary complex apparently reduced the production of EVs (Figure 1). The ESAT-6 epitopes on the EVs were quantified by a two-step immunostaining method using rabbit anti-ESAT-6 polyclonal antibody in the first step, followed by fluorescence-labeling with FITC-goat anti-rabbit IgG antibody. The presentation level of the epitopes, to which the anti-ESAT-6 antibody could bind, was different depending on the transfection method. The number of the epitopes on the EVs from the cells treated with the binary complex was about 12 per particle, while those on the EVs from the cells treated with the ternary complex was around 24 (Figure 2). Transfection with green fluorescent protein (GFP) was carried out simultaneously under similar conditions to compare the foreign gene expression efficiencies of binary and ternary complexes. Gene expression efficiency was evaluated based on the fluorescence intensity of the wells. As shown in Figure 3, the DNA/PEI/CS ternary complex induced 43% to 57% higher levels of reporter protein expression than the DNA/PEI binary complex through 4 days post transfection. As a result, the EVs prepared using the ternary complex were selected for further experiments. As shown in Figure 4, the average particle sizes of ESAT-EV and Naive EV were about 150 nm, with almost identical size distributions.

### 3.2. Activation of DCs by ESAT-EV

**Figure 5:**
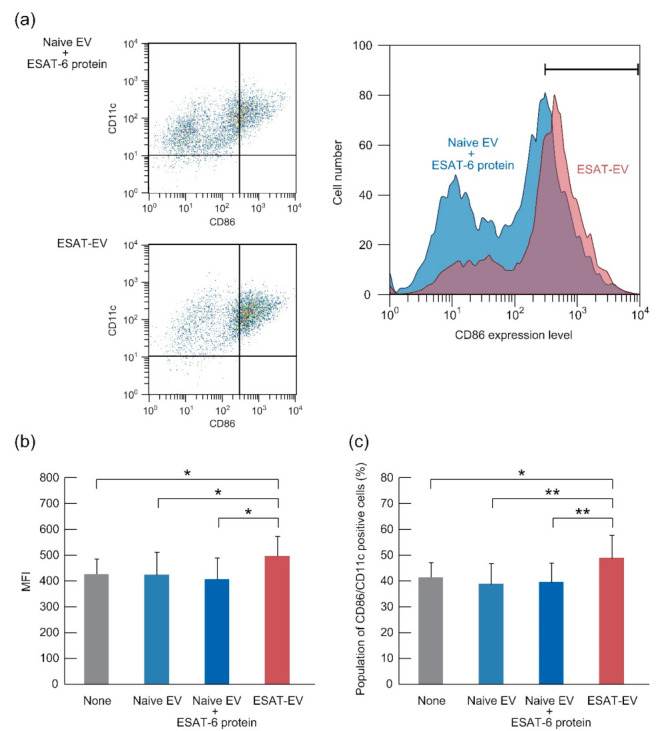
Expression of CD86 by cultured dendritic cells (DCs) treated with ESAT-EV, Naive EV, or a mixture of Naive EV and ESAT-6 protein. DCs were differentiated from the bone marrow cells of C57BL/6 mice, and incubated with ESAT-EV, Naive EV, or a mixture of Naive EV and ESAT-6 protein. After one day of incubation, CD86 protein expressed in the cells was analyzed with a flow cytometer. (**a**) Left, representative dot plots, and right, overlaid histograms of the fluorescence intensity of CD86 in CD11c positive cells treated by ESAT-EV (red) or Naive EV + ESAT-6 protein (blue); (**b**) Mean fluorescence intensity of CD86 in CD11c positive cells (*n* = 3, mean ± SD, * *p* < 0.05); (**c**) Population of the cells with a fluorescence intensity of PE-Cy7-CD86 over 300 counts (*n* = 3, mean ± SD, * *p* < 0.05 and ** *p* < 0.01).

EVs were added to cultured DCs differentiated from the mouse bone marrow cells. Then, the fluorescence labeled anti-CD86 antibody, bound to the DCs, was accessed by FACS. Naive EV or a mixture of Naive EV and ESAT-6 protein did not enhance the mean fluorescence intensity (MFI) or the population of CD86/CD11c positive cells, while ESAT-EV significantly enhanced both values, up to 116% and 119%, respectively (Figure 5).

### 3.3. Antitumor Immune Activation by ESAT-EV

**Figure 6:**
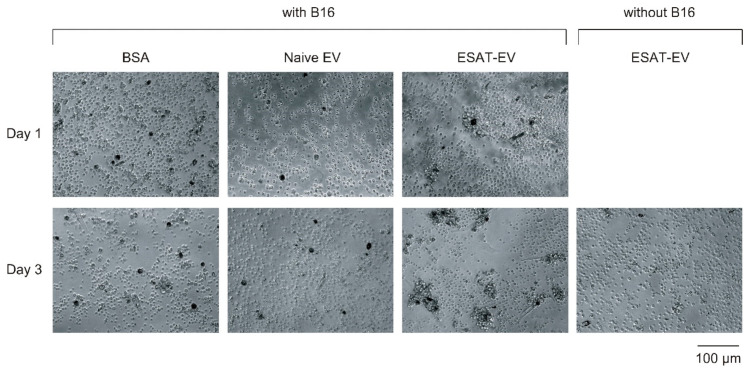
Microscopic images of leukocytes from EV-treated mice incubated with B16 cells. Suspension of ESAT-EV, Naive EV, or BSA solution was injected twice into mouse foot pads on Days 0 and 10. On Day 20, the cells in popliteal lymph nodes were collected, and co-cultured with EV-parent B16 cells pretreated with Mitomycin C at the cell number ratio of leukocyte/B16 = 20:1. Images were taken after 1 or 3 days of co-culture.

**Figure 7:**
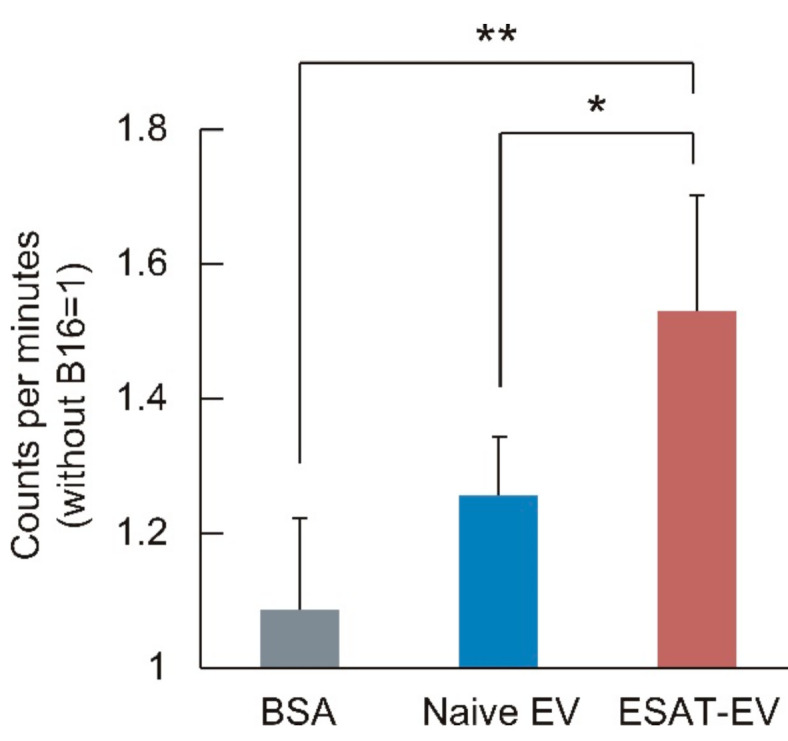
Thymidine incorporation by the leukocytes from EV-treated mice after co-cultured with B16 cells. Leukocytes in the popliteal lymph nodes were collected from mice treated with ESAT-EV, Naive EV, or BSA, and co-cultured with Mitomycin C pretreated B16 cells for 3 days. ^3^H-thymidine was then added, and after overnight incubation, incorporation of the nucleoside into the lymphocyte was measured (*n* = 4, mean ± SD, * *p* < 0.05 and ** *p* < 0.01).

The ability of EVs to induce an immune response against the parent tumor cells was examined. ESAT-EV, Naive EV, or BSA were injected twice into mouse foot pads. Leukocytes were harvested from the dominant lymph nodes, and co-incubated with the EV parent B16 cells. The leukocytes from the mice received an injection of ESAT-EV intensely accumulated around the B16 cells, while the leukocytes from the mice treated with Naive EV or BSA did not show such an accumulation but stayed away from the tumor cells (Figure 6). Leukocytes from ESAT-EV-treated mice exhibited a significantly higher incorporation of 3H-thymidine after the co-incubation with the tumor cells than those collected from Naive EV- or BSA-treated mice (Figure 7). 

### 3.4. Antitumor Therapeutic Efficacy of DCs Treated with ESAT-EV or Naive EV in Tumor-Model Mice

**Figure 8:**
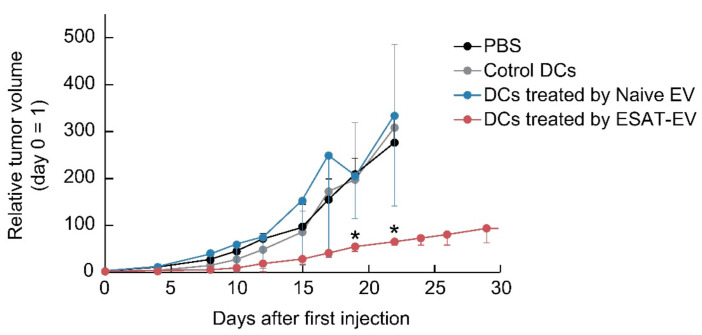
Anti-tumor therapeutic efficacy of DCs treated by EVs. Bone marrow-derived DCs were treated with ESAT-EV, Naive EV, or PBS (as control DCs) for 24 h. Mice bearing B16 tumor were injected intratumorally with the DCs, and the tumor growth was monitored. The tumor volume is calculated as (a × b^2^)/2, where a is the largest and b is the smallest diameter of the tumor and expressed as the ratio between the tumor volume in each time point and at the beginning of the treatment (*n* = 4, mean ± SD, * *p* < 0.05 vs. DCs treated by Naive EV).

The anti-tumor efficacy of DCs stimulated with ESAT-EV in the syngeneic tumor model mice was examined, along with those treated with Naive EV. Mouse bone marrow-derived DCs were co-incubated with ESAT-EV or Naive EV for 24 h. Then, the DCs were washed and injected intratumorally into tumor-bearing mice. On the one hand, the mice that were administered DCs treated with ESAT-EV demonstrated significant suppression of tumor growth, as shown in Figure 8. On the other hand, the mice that received DCs treated with Naive EV or control DCs treated with PBS showed no apparent difference in the tumor growth from that of control mice that received an injection of PBS.

## 4. Discussion

Cultured B16 cells were transfected with the ESAT-6 gene to produce the EVs presenting a Mycobacterium tuberculosis antigen, ESAT-6 (ESAT-EV). In vitro transfection was carried out using a ternary plasmid complex system consisting of pDNA, PEI, and chondroitin sulfate (CS), or by a conventional pDNA/PEI binary complex. We developed the pDNA/PEI/polyanion ternary complex system in order to improve the *in vivo* transfection efficiency. The addition of polyanions, such as hyaluronic acid (HA) or CS, enabled the preparation of very fine particles (70 nm), which realized high in vivo transfection efficiency, especially in tumor tissues after intratumor or intravenous injection [14,15]. On the basis of the research on these DNA ternary complexes, we found that the polyanions could improve the transcription efficiency of the pDNA/PEI complex [16] and invited more prolonged levels of high gene expression on the cells than common DNA/polycation binary complexes [17]. A more durable expression and a lower cell damage would be advantageous for the preparation of transformed cells for the EV preparation. On the one hand, the EV yield of cells transfected with pDNA/PEI/CS ternary complexes was almost the same level as in non-transfected intact cells (Naive EV), and slightly higher than that in binary complex-treated cells. However, no significant differences were detected among them (Figure 1). On the other hand, the ESAT-6 epitope-inducing efficiency was significantly improved by the ternary transfection system. After treatment with anti-ESAT-6 polyclonal antibody, only a small amount (1 molecule per EV particle) of the antibody was detected in Naive EV, without ESAT-6 epitopes on their surfaces, which could be attributed to the non-specific binding of the first or second antibodies. Quantitative fluorescence analysis revealed that the ternary complex transfection system induced significantly higher amounts of ESAT-6 epitopes than the binary complex (Figure 2). A GFP transfection efficiency assay showed that the plasmid/PEI/CS ternary complex exhibited an expression of the reporter gene that was approximately 1.5-fold higher from 1–4 days after transfection (Figure 3). During the preparation of the EVs, the ternary complex system would induce a higher production of the foreign ESAT-6 protein, leading to the secretion of EVs with a higher level of epitope presentation.

ESAT-EV carrying foreign epitopes was expected to potentially stimulate DCs to mature and induce immune responses. DCs were differentiated from bone marrow and co-incubated with the EVs. Naive EV, not presenting an artificial neoepitope, had no effect on the CD86 expression. The addition of the highly immunogenic protein, ESAT-6, to Naive EV did not improve the stimulation ability. However, ESAT-EV, which was expected to present ESAT-6 epitope in the context of MHC class I, induced a significantly higher expression of the DC maturation marker, CD86.

In recent years, DC activation using tumor-derived EVs has been attempted by other research groups, with some favorable results reported. For example, Marton et al. described that CD4+ T cell proliferation could be induced by DCs pretreated with EVs derived from B16F1 melanoma cell culture supernatant [18]. Bu et al. showed that glioma-derived EVs could stimulate DCs to prime CD8+ T-lymphocytes against parent glioma cells [19]. However, the efficacy of simply using tumor-derived EVs from unmodified tumor cells has not always been satisfactory. As mentioned above, tumor-derived EVs can function to both stimulate and suppress the immune response of DCs. A stimulatory molecule would be required to efficiently shift the balance to favor activation. Further improvement was, then, applied on the EVs to enhance the potential to induce DC maturation. Wang et al. prepared EVs carrying the CD40 ligand from 3LL Lewis lung tumor cells by infection with an adenovirus expressing the CD40 ligand. Those EVs induced a more mature phenotype of DCs [20]. The IL-12-anchored exosomes were also prepared by Zhang et al. from RC-2 cells, a human renal cancer cell line. They transfected the cells with a plasmid coding for glycolipid-anchored-IL-12. DCs loaded with the EVs promoted the activation of autologous T cells and elicited more potent cytotoxic effect against the renal cancer cells [21].

In our studies, we employed a strong Mycobacterium tuberculosis antigen, ESAT-6, as an immune-stimulating agent. This microbial antigen was expected to work as an “artificial neoantigen” to induce potent immune responses. Formerly, we induced the antigen by the direct in vivo transfection using an ESAT-6-presenting plasmid. The intratumoral injection of the plasmid complex into tumor bearing mice caused a strong antitumor effect, as well as the induction of cytokine genes [22]. High levels of TNF-α and IFN-γ expression were observed in the tumor tissues [23]. Taking into account the small population of the transfected cells (approximately 10%), the ESAT-6 gene-inducing effect was not attributed to “tumor cell marking”, but likely through the mechanisms mediated by EVs secreted from the ESAT-6-expressing tumor cells. In a previous study, we confirmed that injection of ESAT-EV presenting ESAT-6 epitopes could significantly suppress tumor growth in mice (*p* < 0.001) [13].

As mentioned above, ESAT-EV could effectively activate DCs. Therefore, tumor rejection induced by the injection of ESAT-EV (Figure S1) would be, at least partly, modulated by DCs stimulated by the EVs. If the EV-stimulated DCs could initiate and regulate immune responses against tumors, the administration of EV-treated DCs could also induce similar antitumor action as the injection of EVs in mice carrying tumors. An attempt was made to examine the in vivo antitumor activity of DCs pretreated with ESAT-EV. As shown in Figure 8, DCs pretreated with ESAT-EV demonstrated a significantly improved antitumor efficacy (*p* < 0.001), while non-treated control DCs and Naive EV-treated DCs showed no effect on tumor growth. Because of their origins, EVs, especially released by the autologous cells, were expected to be safe therapeutic agents. Recently, a few clinical trials using autologous EVs have been performed and, thus far, no serious adverse reactions have been reported. It is now widely accepted that administering EVs has high feasibility and safety and low toxicity. However, on the one hand, EV-based therapy does not have a long history, and currently no EV product has been approved for use in humans. On the other hand, DC therapy has been used over the years to treat patients with diverse cancers and has been accepted as safe and feasible. Administration of the purified DCs which were stimulated by the EVs but not contain free EVs would represent a potential alternative and a highly safe method. Unfavorable effects of the TAAs on the tumor-derived EVs were also reported. Treatment with tumor-derived EVs carrying TAAs interfered with the antigen-specific recognition of tumor cells by antitumor antibodies or tumor-specific cytotoxic T lymphocytes (CTLs) [5]. Those adverse effects should be avoided by the treatment with the EV-stimulated DCs not containing free EVs.

## 5. Conclusions

Tumor-derived EVs presenting a highly immunogenic bacterial antigen, ESAT-6, was prepared by genetically modifying the parent B16 tumor cells with a plasmid coding for ESAT-6. The ESAT-6-presenting EVs stimulated cultured DCs to induce high CD86 production. They elicited an evident immune response against the parent tumor cells in normal mice. Injection of the DCs which had been pre-stimulated by the EVs showed significantly improved antitumor activity in tumor-bearing mice.

Our study indicates that microbial antigen-presenting tumor-derived extracellular vesicles could efficiently provide activating signals to DCs to cross-prime tumor-directed CTLs. It may represent a novel strategy leading to clinical responses, even for tumors with low immunogenicities.

## Data Availability

The data presented in this study are available on request from the corresponding author.

## Supplementary Materials

The following are available online at https://www.mdpi.com/1999-4923/13/1/57/s1, Figure S1: Anti-tumor effect of ESAT-EV and Naive EV in tumor-bearing mice. B16 cells were inoculated subcutaneously into C57BL/6 mice (10^6^ cells per mouse). When the major axis of the tumor reached 4–7 mm, ESAT-EV (3.2 × 10^9^ vesicles), Naive EV (3.3 × 10^9^ vesicles), a mixture of Naive EV and 0.1 μg ESAT-6 protein, or PBS, was injected intratumorally three times at 4 days interval (*n* = 5, mean ± SD, *** *p* < 0.001 vs. Naive EV).
