# Supplementary material for: Microbial Antigen-Presenting Extracellular Vesicles Derived from Genetically Modified Tumor Cells Promote Antitumor Activity of Dendritic Cells
Source: Pharmaceutics. 2021 Jan 4;13(1):57. doi: 10.3390/pharmaceutics13010057 (PMC7824503; doi:10.3390/pharmaceutics13010057)
Supplement: Supplementary file 1 [file pharmaceutics-13-00057-s001.pdf]

pharmaceutics

Supplementary Materials: Microbial Antigen-Presenting Extracellular Vesicles Derived from Genetically Modified Tumor Cells Promote Antitumor Activity of Dendritic Cells

Tomoko Ito, Kikuya Sugiura, Aya Hasegawa, Wakana Ouchi, Takayuki Yoshimoto, Izuru Mizoguchi, Toshio Inaba, Katsuyuki Hamada, Masazumi Eriguchi and Yoshiyuki Koyama

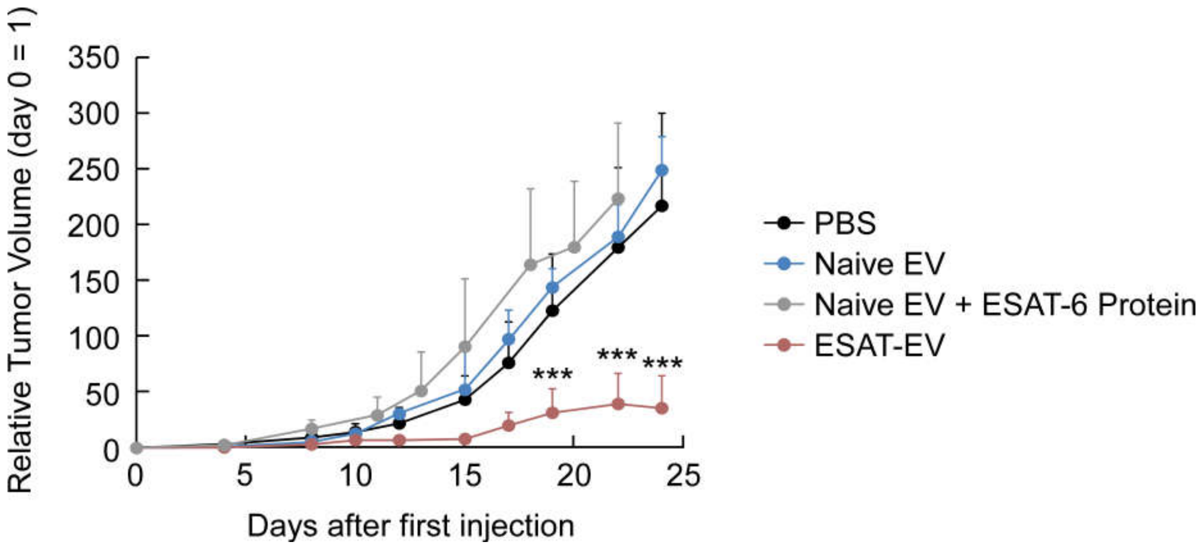

Figure S1. Anti-tumor effect of ESAT-EV and naive EV in tumor-bearing mice. B16 cells were inoculated subcutaneously into C57BL/6 mice (10^6 cells per mouse). When the major axis of the tumor reached 4–7 mm, ESAT-EV (3.2×10^9 vesicles), naive EV (3.3×10^9 vesicles), a mixture of naive EV and 0.1 µg ESAT-6 protein, or PBS, was injected intratumorally three times at 4 days interval ($n = 5$, mean ± SD, *** $p < 0.001$ vs. naive EV).
